# Effects of Pera Orange Juice and Moro Orange Juice in Healthy Rats: A Metabolomic Approach

**DOI:** 10.3390/metabo13080902

**Published:** 2023-08-02

**Authors:** Anderson S. S. Fujimori, Ana P. D. Ribeiro, Amanda G. Pereira, Flávia L. Dias-Audibert, Carolina R. Tonon, Priscila P. dos Santos, Danielle Dantas, Silmeia G. Zanati, Rodrigo R. Catharino, Leonardo A. M. Zornoff, Paula S. Azevedo, Sergio A. R. de Paiva, Marina P. Okoshi, Estela O. Lima, Bertha F. Polegato

**Affiliations:** 1 Internal Medicine Department, Botucatu Medical School, São Paulo State University (UNESP), Botucatu 18618-687, Brazil; seiji.fujimori@unesp.br (A.S.S.F.); ana.d.ribeiro@unesp.br (A.P.D.R.); ag.pereira@unesp.br (A.G.P.); carolina.tonon@unesp.br (C.R.T.); priscila.portugal@unesp.br (P.P.d.S.); d.dantas@unesp.br (D.D.); sgz.bazan@unesp.br (S.G.Z.); leonardo.zornoff@unesp.br (L.A.M.Z.); schmidt.azevedo@unesp.br (P.S.A.); sergio.paiva@unesp.br (S.A.R.d.P.); marina.okoshi@unesp.br (M.P.O.); estela.lima@unesp.br (E.O.L.); 2 Innovare Biomarkers Laboratory, School of Pharmaceutical Sciences, University of Campinas, Campinas 13083-970, Brazil; flaviald.nutricao@gmail.com (F.L.D.-A.); rrc@unicamp.br (R.R.C.)

**Keywords:** *Citrus sinensis* L. Osbeck, cardiac remodeling, extracellular matrix metalloproteinases, inflammation, cardiac function

## Abstract

Cardiovascular disease is a leading cause of death worldwide. Heart failure is a cardiovascular disease with high prevalence, morbidity, and mortality. Several natural compounds have been studied for attenuating pathological cardiac remodeling. Orange juice has been associated with cardiovascular disease prevention by attenuating oxidative stress. However, most studies have evaluated isolated phytochemicals rather than whole orange juice and usually under pathological conditions. In this study, we evaluated plasma metabolomics in healthy rats receiving Pera or Moro orange juice to identify possible metabolic pathways and their effects on the heart. Methods: Sixty male Wistar rats were allocated into 3 groups: control (C), Pera orange juice (PO), and Moro orange juice (MO). PO and MO groups received Pera orange juice or Moro orange juice, respectively, and C received water with maltodextrin (100 g/L). Echocardiogram and euthanasia were performed after 4 weeks. Plasma metabolomic analysis was performed by high-resolution mass spectrometry. Type I collagen was evaluated in picrosirius red-stained slides and matrix metalloproteinase (MMP)-2 activity by zymography. MMP-9, tissue inhibitor of metalloproteinase (TIMP)-2, TIMP-4, type I collagen, and TNF-α protein expression were evaluated by Western blotting. Results: We differentially identified three metabolites in PO (N-docosahexaenoyl-phenylalanine, diglyceride, and phosphatidylethanolamine) and six in MO (N-formylmaleamic acid, N2-acetyl-L-ornithine, casegravol isovalerate, abscisic alcohol 11-glucoside, cyclic phosphatidic acid, and torvoside C), compared to controls, which are recognized for their possible roles in cardiac remodeling, such as extracellular matrix regulation, inflammation, oxidative stress, and membrane integrity. Cardiac function, collagen level, MMP-2 activity, and MMP-9, TIMP-2, TIMP-4, type I collagen, and TNF-α protein expression did not differ between groups. Conclusion: Ingestion of Pera and Moro orange juice induces changes in plasma metabolites related to the regulation of extracellular matrix, inflammation, oxidative stress, and membrane integrity in healthy rats. Moro orange juice induces a larger number of differentially expressed metabolites than Pera orange juice. Alterations in plasma metabolomics induced by both orange juice are not associated with modifications in cardiac extracellular matrix components. Our results allow us to postulate that orange juice may have beneficial effects on pathological cardiac remodeling.

## 1. Introduction

Cardiovascular disease (CVD) is a leading cause of death worldwide [1,2]. Within CVD, heart failure (HF) has a high prevalence and represents a burden to health systems and patients due to its elevated morbidity and mortality [3]. HF is a complex and multifactorial syndrome associated with cardiac remodeling [3,4]. Cardiac remodeling is characterized by molecular, cellular, and interstitial changes that manifest as changes in heart size, geometry, and function following various injury etiologies [5]. Several mechanisms are involved in the pathophysiology of cardiac remodeling and heart failure development; these include neurohormonal activation, inflammation, cell death, contractile protein changes, metalloproteinases regulation, fibrosis, extracellular matrix alterations, and oxidative stress [4].

It is estimated that up to 75% of premature CVD may be prevented or even treated with healthy lifestyle choices, especially dietary patterns [6]. Regular consumption of food rich in bioactive compounds has been associated with improved cardiometabolic health, probably due to their antioxidant and anti-inflammatory properties [7,8].

Orange juice, which is a dietary-rich source of ascorbic acid, carotenoids, phenolic acids, and flavonoids has been associated with the prevention of CVD [9,10,11,12]. We have recently observed doxorubicin-induced cardiotoxicity attenuation [13] and improvements in oxidative stress after myocardial infarction [14] in rats that received orange juice. Additionally, studies have shown that orange juice improves liver damage in obese rats, and metabolism and clearance of blood lipoprotein particles in normolipidemic rats [15]. Two recent studies have shown a reduction in plasma glucose, cholesterol, and low-density lipoprotein [16], and beneficial changes in transcriptomics [17] in hypertensive patients receiving orange juice. 

Oranges are the most cultivated fruit in the world and make up 0.9% of total world fruit production. The Pera orange (*Citrus sinensis* L. Osbeck var. Pera-Rio) is the variety most commonly used for juice extraction. More recently, a type of blood orange (*Citrus sinensis* L. Osbeck var. Moro) named Moro has been investigated for its health-related properties [18,19]. Its typical red coloration is attributed to the presence of anthocyanins, pigments with high antioxidant and anti-inflammatory properties not usually found in sweet blond oranges [19]. Additionally, this type of orange has higher levels of ascorbic acid and hesperidin, thus providing more antioxidant and anti-inflammatory properties than Pera orange juice [20]. 

Traditionally, nutritional research is based on identifying specific compounds [21]. For example, eicosapentaenoic acid is often used in fish oil studies to describe the polyunsaturated fatty acid group, but other potential compounds are not analyzed [21]. More recently, integrative metabolomics has emerged as a science that provides a quantitative and qualitative characterization of thousands of metabolites and nutrients simultaneously in a biological sample [22]. It is a promising field of nutritional research which allows the integrated and complete evaluation of specific molecules and their metabolic pathways, as well as inter-individual variability in metabolizing foods in health and disease situations [23]. 

Few studies have used metabolomics to evaluate the effects of orange juice ingestion. Moreira et al. showed that a two-week period of orange intake affected peroxisomal and mitochondrial fatty acid β-oxidation in plasma from healthy volunteers and Rangel-Huerta et al. reported that consumption of orange juice with different polyphenol levels modulated inflammation and oxidative stress-related metabolites in overweight and obese individuals [24,25]. In this study, we evaluated the effects of Pera and Moro orange juice administration in healthy rats by plasma metabolomics. After metabolomic analysis, we differentially identified three metabolites in serum of rats that received Pera orange juice and six metabolites in rats treated with Moro orange juice. Since these metabolites are related to extracellular matrix regulation, we performed morphological and biochemical myocardial assessments to verify the influence of orange juice on myocardium from healthy animals.

## 2. Materials and Methods

### 2.1. Study Design

**Figure 1:**
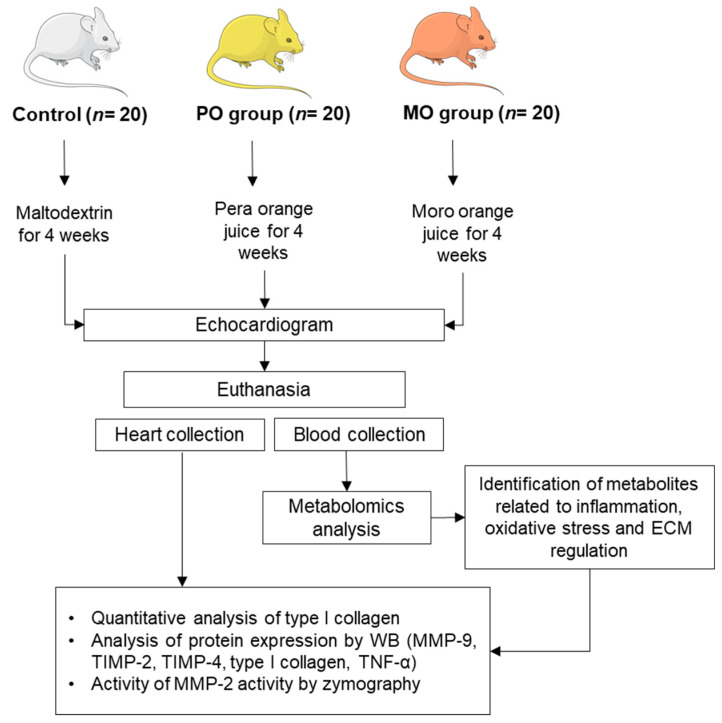
Flowchart of experimental protocol. PO: Pera orange juice; MO: Moro orange juice; ECM: extracellular matrix; WB: Western blot; MMP: matrix metalloproteinase, TIMP: tissue inhibitor of metalloproteinase.

This study was approved by our local Ethics Committee (protocol nº 1285/2019) and was performed in accordance with the National Council for Animal Experiment Control standards. Sixty male Wistar rats weighing 250 to 300 g were allocated into 3 groups of 20 rats each: control (C), Pera orange juice (PO), and Moro orange juice (MO). The rats were kept in a controlled environment with a 12 h light/dark cycle at 23 ± 2 °C and free access to regular chow. PO and MO groups received pasteurized Pera orange juice or Moro orange juice ad libitum, respectively. Controls received water with maltodextrin (100 g/L) to match juice carbohydrate level. The experimental period was 4 weeks. Liquid intake was measured throughout the experiment as the difference between daily offered volume (100 mL) and amount remaining in bottles after 24 h (Figure 1). Juices were kindly donated by Fundecitrus (Fundo de Defesa da Citricultura, Araraquara, SP, Brazil). At the end of the experiment, rats underwent echocardiogram and were euthanized the next day after anaesthesia with sodium thiopental, 120 mg/kg, intraperitoneal (IP). Hearts and blood were collected. The hearts were washed in fresh saline, dissected, weighed, and stored at -80 °C. Blood was collected and centrifuged; serum was stored at −80 °C.

### 2.2. High-Resolution Mass Spectrometry

Rat serum samples were subjected to analytical techniques to assess metabolomic profile. Serum samples were prepared for five replication analysis according to Melo et al. [26]. Samples were subsequently ionized by electrospray and subjected to high-resolution mass spectrometry (ESI-HRMS), in an ESI-LTQ-XL Orbitrap Discovery Analyzer (Thermo Scientific, Bremen, Germany), with a nominal resolution equivalent to 30,000 (FWHM). Two mass ranges were defined for our analysis, 100–700 *m*/*z* and 700–1700 *m*/*z*, both in positive and negative mode of analysis, and spectra were analyzed using XCalibur Version 2.4 (Thermo Scientific, San Jose, CA, USA). Biomarker identification and structural elucidation were performed based on comparing experimental and theoretical masses using online databases, such as METLIN (Scripps Center for Metabolomics, La Jolla, CA, USA). Initial analysis suggested some differential biomarker expression between PO and C and MO and C groups, which was based on mass accuracy in ppm. The most relevant biomarkers were selected. Finally, identification of the selected biomarkers was confirmed based on the ion fragmentation profile in tandem (MS/MS), using Mass Frontier software Version 6.0 (ThermoScientific, San Jose, CA, USA).

### 2.3. Echocardiogram

Rats were anesthetized with ketamine (50 mg/kg, IP) and xylazine (1 mg/kg, IP), and subjected to echocardiogram using a commercially available echocardiograph (General Electric Medical Systems, Vivid S6 model, Israel) with a 5.0–11.5 MHz multifrequency transducer using a previously described method [27]. The following structural variables were obtained by two-dimensional analysis: left atrium diameter (LA), LV diastolic and systolic diameters (LVDD and LVSD, respectively), left ventricular (LV) diastolic and systolic posterior wall thickness (PWT), septal wall thickness (SWT), and interventricular septum thickness (IVST). Function was assessed by early and late diastolic mitral inflow velocities (E and A waves), E/A ratio, E-wave deceleration time (EDT), and isovolumetric relaxation time (IVRT). The evaluation was complemented by tissue Doppler imaging (TDI) of systolic (S’), early diastolic (E’), and late diastolic (A’) velocity of the mitral annulus. Data were used to calculate LV shortening fraction [(LVDD-LVSD)/LVDD] and E/E’ ratio.

### 2.4. Quantitative Analysis of Type I Collagen in Histological Sections

After euthanasia, a 2 mm thick cross-section ring of LV was obtained 5 mm from the LV apex. The ring was placed in 10% buffered formaldehyde solution for 24 to 48 h and then transferred to 70% ethanol solution until processing. These were then embedded in paraffin block, and 5 μm thick sections were cut with a microtome and stained with picrosirius red. The sections then underwent optical density examination and quantification of collagen fiber content using Image Pro–plus (Media Cybernetics, Rockville, MD, USA) software (31).

### 2.5. MMP-2 Activity

Approximately 30 mg of LV tissue was added to extraction buffer (Tris 50 mM pH 7.4, NaCl 0.2 M, Triton X 0.1%, and CaCl_2_ 10 mM), crushed and centrifuged. Protein was quantified in supernatant by the Bradford method [28]. Samples (10 μg of protein) were diluted in sample buffer (Tris 0.5 M pH 6.8, glycerol 50% and bromophenol blue 0.05%) and submitted to electrophoresis on 8% polyacrylamide plus 1% gelatin gels. After running, the gels were washed with Triton X-100 2.5% and Tris-HCl 50 mM pH 8.4 and incubated for 17 h at 37 °C with continuous agitation (tris-HCl 50 mM pH 8.4, CaCl_2_ 500 mM buffer). Subsequently, gels were stained with Coomassie brilliant blue 2.5% and discolored by 30% methanol and 10% acetic acid. A control sample was included in each gel for normalizing results. Matrix metalloproteinase (MMP)-2 position in the gels was confirmed by recombinant rat/mouse MMP-2 standard (R&D Systems, Minneapolis, MN, USA). The gels were photographed by ImageQuant LAS (General Electrics Healthcare, Arlington Heights, IL, USA) and analyzed by Gel-Pro 3.2 (Media Cybernetics, Rockville, MD, USA).

### 2.6. Protein Expression

Samples of LV tissue (100 mg) were homogenized in RIPA buffer and centrifuged. Supernatants were collected and total protein quantified by the Bradford method. Electrophoresis was performed in acrylamide gels and proteins transferred to nitrocellulose membranes, which were incubated in 5% skimmed milk. Then, membranes were incubated with primary antibodies anti-matrix metalloproteinase (MMP-9), tissue inhibitor of metalloproteinase (TIMP)-2, TIMP-4, type I collagen, and TNF-α (SC-393859, Santa Cruz Biotechnology Inc., Dallas, TX, USA; SC-21735, Santa Cruz Biotechnology Inc., Dallas, TX, USA; BOST-PA1078, Boster Biological Technology, Pleasanton, CA, USA; SC-293182, Santa Cruz Biotechnology Inc., Dallas, TX, USA; SC-52746, Santa Cruz Biotechnology Inc., Dallas, TX, USA, respectively) for 12 h, and then incubated with secondary antibodies. Antibody anti-GAPDH (SC-32233, Santa Cruz Biotechnology Inc., Dallas, TX, USA) was used for normalization.

### 2.7. Statistical Analysis

Data are expressed as mean ± SD or median and lower and upper quartiles. Comparisons between groups were performed by one-way ANOVA or ANOVA on ranks complemented by the Tukey test. A statistical significance of 5% was adopted. For metabolomic analysis, principal component analysis (PCA) and partial least squares discriminant analysis (PLS-DA) data were evaluated by multivariate statistics. Through this analysis, we were able to identify molecules which had different expressions between the PO and C or MO and C groups. Selection of each biomarker was based on its importance for the model evaluated, which was identified using the variable importance in projection (VIP) score. From the most important biomarkers, a heatmap was designed using Euclidean distance measurement and the Ward’s clustering method. PLS-DA, VIP score analysis, and heatmap design were performed using MetaboAnalyst 3 software.

## 3. Results

### 3.1. Body Weight and Fluid Intake

Body weight at the end of the experimental period did not differ between groups [C 368 (334–392); PO 365 (320–398); MO 354 (335–367) g; *p* = 0.478]. Daily fluid intake was lower in MO than in C and did not differ between PO and C groups [C 67.7 (57.8–68.5); PO 64.9 (57.4–66.4); MO 62.8 (56.1–66.6) mL; *p* = 0.029]. 

### 3.2. High-Resolution Mass Spectrometry

**Figure 2:**
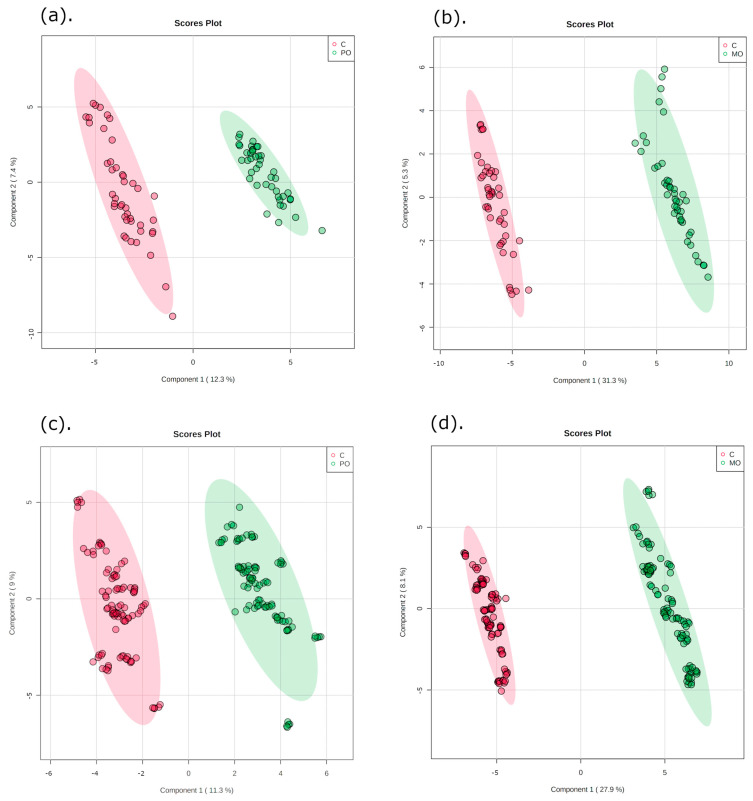
Partial least squares discriminant analysis (PLS-DA) of orange juice ingestion (green) and control groups (red) for two mass ranges. Graphics (**a**,**b**): *m*/*z* = 100–700; graphics (**c**,**d**): *m*/*z* = 700–1700. C: control; PO: Pera orange juice; MO: Moro orange juice.

**Figure 3:**
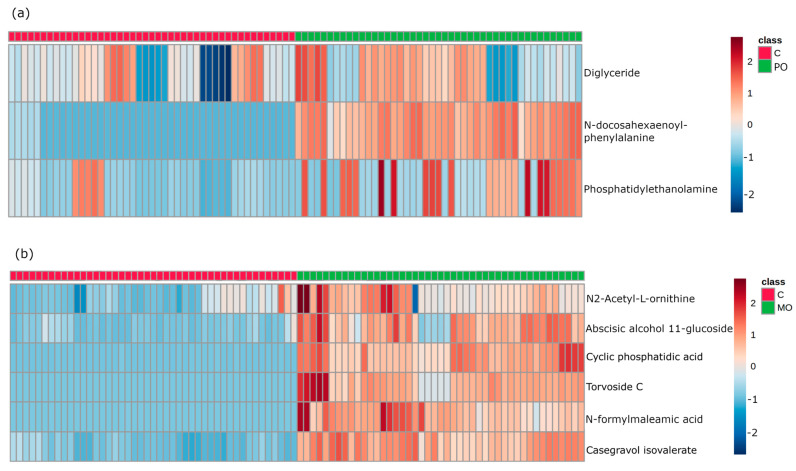
Heatmap of important features from variable importance in projection (VIP) score in partial least squares discriminant analysis (PLS-DA). Heatmap analysis of the most important biomarkers elected through PLS-DA. Metabolites are displayed in rows and the samples are represented in columns (■ control samples (CT); ■ orange juice samples). Colors indicate metabolite intensity for each sample, where darker red indicates a biomarker’s higher intensity and darker blue a biomarker’s lower intensity. (**a**): identification of metabolites in control and Pera orange (PO) groups; (**b**): identification of metabolites in control and Moro orange (MO) groups.

The multivariate data analysis PLS-DA was performed, comparing *m*/*z* values and precursor ion intensities between MO and C, and between PO and C groups, separated into two mass ranges: *m*/*z* = 100–700 and *m*/*z* = 700–1700, on positive ion mode, as shown in Figure 2. These analyses generated VIPs for biomarkers according to their importance (Figure S1), which allowed identification of the most important differentially expressed biomarkers for each juice group. These biomarkers were then evaluated for their frequency in each orange juice group compared to controls; these are shown in the heatmap (Figure 3).

After selection, the most important biomarkers were submitted to in tandem mass spectrometry and all metabolite fragments were acquired and analyzed for structural elucidation. Then, by searching well-established metabolite databases, we were able to select and identify three molecules in the PO and six molecules in the MO group which were differentially expressed compared to the C group. Table 1 and Table 2 show the datasets of these molecules identified in PO and MO groups, respectively.

Metabolomic analysis identified some metabolites that are precursors of molecules mostly related to the biological processes involved in modulating inflammation, oxidative stress, and extracellular matrix, which are important regulators of cardiac remodeling [4,29,30,31,32,33]. These metabolites identified in the PO and MO groups may have beneficial effects on cardiac remodeling and prevent heart failure development and progression. From metabolomic analysis, we proceeded to evaluate the myocardial components associated with inflammation and extracellular matrix regulation. 

### 3.3. Echocardiography

Echocardiographic data after 4 weeks of juice intake showed no structural cardiac or LV functional differences between the groups (Table 3).

### 3.4. Myocardial Collagen Content 

**Figure 4:**
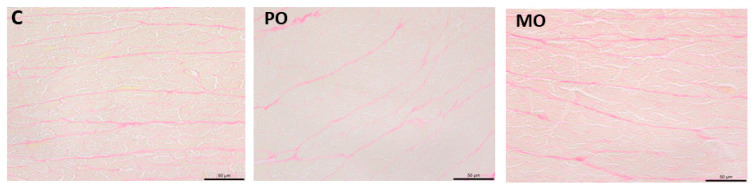
Picrosirius red-stained histological sections. C: control; PO: Pera orange juice; MO: Moro orange juice.

Myocardium was evaluated using picrosirius red-stained histological sections for quantitative collagen analysis. The percentage of collagen tissue did not differ between groups (C 2.33 ± 0.66; PO 2.53 ± 0.58; MO 2.50 ± 0.64%; *p* = 0.744; Figure 4).

### 3.5. Myocardial MMP-2 Activity

**Figure 5:**
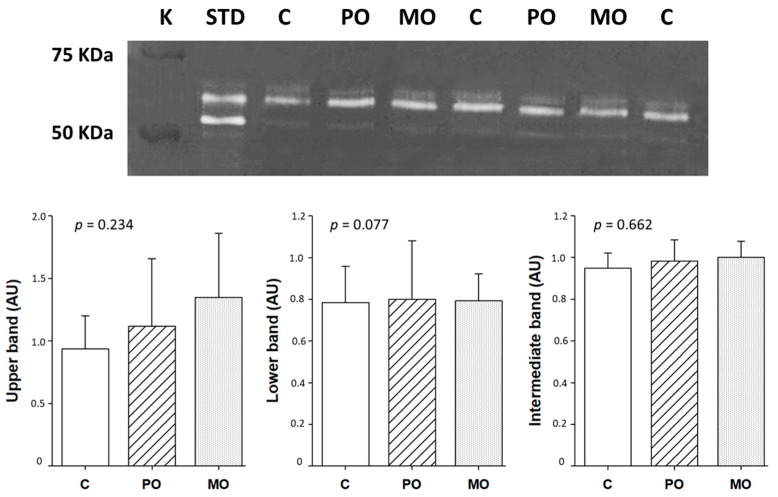
Matrix metalloproteinase (MMP)-2 activity by zymography. White bands represent the position of gelatin digestion in the gel and, consecutively, MMP-2 activity. C: control; PO: Pera orange juice; MO: Moro orange juice; K: kaleidoscope molecular weight standard; STD: positive control (mouse/rat recombinant MMP-2, R&D System, MN, USA); AU: arbitrary units. Data are expressed as mean ± SD; ANOVA and Tukey.

MMP-2 activity did not differ between groups (Figure 5).

### 3.6. Type I Collagen, TIMP-2, TNF-α, MMP-9, and TIMP-4 Expression

Protein expression of type I collagen, TIMP-2, TNF-α, MMP-9, and TIMP-4 did not differ between groups (Table 4).

## 4. Discussion

This is the first study to assess plasma metabolomics in healthy rats that received Pera or Moro orange juice. Using metabolomics, we identified three metabolites in the PO group and six in the MO group that significantly differed from the controls in healthy rat plasma after administration of Pera and Moro orange juice. Metabolomics is an emerging and promising approach for identifying new biomarkers and providing new insights into nutritional science [24], that allows a better understanding of the association between specific substances and metabolic pathways. 

In the past few years, several studies have focused on the role of antioxidants in orange juice, mainly flavonoids (hesperidin and naringenin), carotenoids (xanthophylls, cryptoxanthins, carotenes), and vitamin C as contributors in preventing or treating CVD and other chronic illnesses. However, most studies have evaluated isolated phytochemicals from the orange juice instead of whole juice intake, and focused on pathological conditions [34,35,36,37].

We identified differentially expressed biomarkers according to their mass/charge (*m*/*z*) ratios. Metabolites in the PO group corresponded to: N-docosahexaenoyl-phenylalanine (*m*/*z* 476.3168), diglyceride (DG; 20:4/24:1) (*m*/*z* 709.6141), and phosphatidylethanolamine (PE; O-20:0/16:0) (*m*/*z* 734.6044). The first metabolite is a phenylalanine-derived compound, a lipid signaling molecule. Its activity is not completely understood, but seems related to analgesic and anti-inflammatory action [38,39], indicating that it could be an interesting target in cardiac remodeling [29,40]. DG and PE are involved in phospholipid biosynthesis [41] such as lipid transportation, metabolism, and peroxidation. Diacylglycerols are important players in modulating membrane fluidity [42]. PE is one of the most abundant glycerophospholipids in the cell membrane, and essential for its integrity, particularly in the human heart. After myocardial infarction, the main phospholipids significantly decreased in the LV, indicating phospholipolysis [43]. Together, the presence of these biomarkers in the plasma metabolomics of rats that received Pera orange juice suggests a possible protective effect on cell membranes. 

We identified six differentially expressed molecules in MO compared to controls: N-formylmaleamic acid (*m*/*z* 181.9853), N2-acetyl-L-ornithine (*m*/*z* 197.0893), casegravol isovalerate (*m*/*z* 361.1639), abscisic alcohol 11-glucoside (*m*/*z* 435.1981), cyclic phosphatidic acid (CPA; 18:2) (*m*/*z* 455.1968), and torvoside C (*m*/*z* 779.3994). N-formylmaleamic acid is a metabolite of nicotinamide, also known as Vitamin B3, which modulates inflammation, cell signaling, and the synthesis of both nicotinamide adenine dinucleotide (NAD+/NADH) and triphosphopyridine nucleotide (NADP+/NADPH), with a role in energy metabolism and oxidative status [44,45,46]. Increased oxidative stress and inflammation stimulates metalloproteinases, impairing cardiac remodeling. Therefore, N-formylmaleamic acid may have antioxidant effects and contribute to cardiac protection. 

N2-acetyl-L-ornithine is a precursor of L-arginine, an essential amino acid related to nitric oxide (NO) [47]. Interestingly, O’Sullivan et al. have shown that NO modulates MMP-9 expression and activity [48]. However, the results are conflicting, since NO seems to act either inhibiting or activating MMP-9 [48]. Thus, as N2-acetyl-L-ornithine can be metabolized to NO, which regulates MMP-9 activity; this molecule may interfere with the dynamics and composition of the ECM.

Casegravol isovalerate, also known as sparoxomycin A1, is a coumarin compound found in plants [49]. It has been used as an anticoagulant, antifungal, and antioxidant [50,51,52]. Another metabolite associated with MO juice consumption was abscisic alcohol 11-glucoside, an intermediate of abscisic acid (ABA) biosynthesis. This molecule is a phytohormone responsible for the synthesis of flavonoids in fruits, including anthocyanins. The biological function of casegravol and abscisic alcohol 11-glucoside remains unknown in both physiological and pathological processes [53,54,55]. 

CPA [18:2] acts in several biological functions, including antimitogenic regulation of the cell cycle, inhibition of tumor cell invasion and metastasis, and regulation of neuronal cell differentiation [56,57,58]. Furthermore, CPA induces hyaluronic acid synthesis in vitro and in vivo [59,60]. Although hyaluronic acid is a major component of the ECM [61], there are no studies related to its role in myocardial ECM. Lastly, torvoside C, also known as steroidal saponin (SE), may modulate ECM and inflammation. In rats with diabetic nephropathy, chronic treatment with SE decreased collagen-IV and fibronectin in the kidney [62,63]. The addition of SE to in vitro prostate cancer cells reduced MMP-2 and MMP-9 activity and the expression of nuclear transcription factor-kappa B, and increased TIMP-2 activity [64]. Also, SE administration decreased TNF-α, interleukin-6, and interleukin-1-β in rats with doxorubicin-induced cardiotoxicity [65]. This evidence suggests that steroidal saponins are promising substances in the treatment of pathologies related to ECM changes and inflammation, such as heart failure.

To the best of our knowledge, we did not identify studies that evaluated the metabolomics after orange juice intake in heart disease models, which could be interesting to characterize the possible protective effect of these metabolites in pathological pathways.

In this study, orange juice induced changes in plasma metabolites related to the regulation of myocardial ECM, inflammation, oxidative stress, and membrane integrity. These alterations were not associated with modifications in components of cardiac ECM in healthy rats. Taken together, our findings allow us to hypothesize that orange juices, especially Moro orange juice, may have beneficial effects in cardiovascular disease in which ECM alteration and inflammation are involved in the pathophysiologic process. Studies involving pathological cardiac remodeling models are needed to clarify this issue.

## 5. Conclusions

Ingestion of Pera and Moro orange juice induces changes in plasma metabolites related to the regulation of myocardial extracellular matrix, inflammation, oxidative stress, and membrane integrity in healthy rats. Moro orange juice induces a larger number of differentially expressed metabolites than Pera orange juice. Alterations induced by both orange juices in plasma metabolomics are not associated with modifications in components of cardiac extracellular matrix.

## Figures and Tables

**Table 1:** Metabolomics biomarkers elected by partial least squares discriminant analysis (PLS-DA) from plasma analysis of Pera orange (PO) group in positive ion mode.

Exact Mass	MS/MS	Theoretical Mass	Adduct	MID ^a^	Molecule	Error (ppm)
476.3168 *	325—572—755—492—843	476.3159	[M + H]^+^	75476	N-docosahexaenoyl-phenylalanine	−1.88
**709.6141 ^#^**	485—493—659—507	709.6135	[M + H-H_2_O]^+^	59046	DG (20:4/24:1)	−0.84
**734.6044 ^#^**	615—703—367—418	734.6058	[M + H]^+^	77570	PE (O-20:0/16:0)	1.90

High-resolution mass spectrometer (HRMS) biomarkers elected by partial least squares discriminant analysis (PLS-DA) associated with variable importance in projection (VIP) score. Identification based on mass spectrometry in tandem and metabolomics databases. MS/MS: ion fragmentation profile; ^a^: METLIN ID; DG: diglyceride; PE: phosphatidylethanolamine; *: mass range from *m*/*z* 100–700 Da; ^#^: mass range from *m*/*z* 700–1700.

**Table 2:** Metabolomics biomarkers elected by partial least squares discriminant analysis (PLS-DA) from plasma analysis in Moro orange (MO) group in positive ion mode.

Exact Mass	MS/MS	Theoretical Mass	Adduct	MID ^a^	Molecule	Error (ppm)
181.9853 *	165—136—112	181.9850	[M + K]^+^	72080	N-formylmaleamic acid	−1.64
197.0893 *	127—134—179—81	197.0897	[M + Na]^+^	3303	N2-Acetyl-L-ornithine	2.02
361.1639 *	305—291—231—213	361.1646	[M + H]^+^	93640	Casegravol isovalerate	1.93
435.1981 *	403—393—365—417	435.1989	[M + Na]^+^	94192	Abscisic alcohol 11-glucoside	1.83
455.1968 *	437—385—315—329	455.1959	[M + K]^+^	58642	CPA (18:2) ^a^	−1.97
**779.3994 ^#^**	659—719—709—733	779.3979	[M + K]^+^	86383	Torvoside C	−1.92

High-resolution mass spectrometer (HRMS) biomarkers elected by partial least squares discriminant analysis (PLS-DA) associated with variable importance in projection (VIP) score. Identification based on mass spectrometry in tandem and metabolomics databases. MS/MS: ion fragmentation profile; ^a^: METLIN ID; CPA: cyclic phosphatidic acid; *: mass ranges from *m*/*z* 100–700; ^#^: mass range from *m*/*z* 700–1700.

**Table 3:** Echocardiographic data.

Variable	C (*n* = 20)	PO (*n* = 20)	MO (*n* = 20)	*p*-Value
HR (bpm)	323 ± 78	334 ± 67	350 ± 60	0.453
PWT (mm)	1.53 (1.45–1.53)	1.53 (1.53–1.53)	1.53 (1.45–1.53)	0.306
IVST (mm)	1.53 (1.53–1.64)	1.53 (1.53–1.58)	1.53 (1.53–1.65)	0.674
LVDD (mm)	6.68 ± 0.59	6.7 8± 0.64	6.71 ± 0.55	0.850
LVSD (mm)	2.60 ± 0.37	2.80 ± 0.5	2.70 ± 0.56	0.486
LA (mm)	4.34 ± 0.31	4.51 ± 0.32	4.47 ± 0.24	0.162
E/A	1.42 (1.25–1.60)	1.46 (1.38–1.62)	1.41 (1.34–1.57)	0.501
E’ (cm/s)	6.21 ± 1.09	6.17 ± 0.86	6.10 ± 0.23	0.947
A’ (cm/s)	3.93 ± 0.47	3.91 ± 0.56	4.11 ± 0.55	0.403
E/E’	14.8 (13.9–15.6)	14.4 (13.2–15.3)	13.8 (13.3–15.6)	0.502
IVRTn (ms)	53.4 (47.9–58.0)	53.6 (50.4–60.3)	57.2 (53.1–59.3)	0.253
EDT (ms)	45.8 ± 6.60	49.6 ± 6.10	48.6 ± 5.20	0.132
S’ (cm/s)	5.82 (5.47–6.00)	5.97 (5.80–6.10)	5.95 (5.72–6.22)	0.351
FS	0.62 (0.58–0.64)	0.59 (0.56–0.62)	0.60 (0.56–0.64)	0.349

C: control; PO: Pera orange juice; MO: Moro orange juice. HR: heart rate; PWT: posterior wall thickness; IVST: interventricular septum thickness; LVDD; left ventricle (LV) diastolic diameter; LVSD: LV systolic diameter; LA: left atrium diameter; E/A: peak velocity of early ventricular filling/peak transmitral flow velocity during atrial contraction; E’: peak early diastolic mitral annulus velocity determined by tissue Doppler imaging (TDI); A’: peak late diastolic mitral annular motion velocity determined by TDI; IVRTn: isovolumetric relaxation time normalized to heart rate; EDT: E-wave deceleration time; S’: peak systolic mitral annular motion velocity determined by TDI; FS: fractional shortening. Data are expressed as mean ± SD or median and 25th and 75th percentiles; ANOVA and Tukey.

**Table 4:** Protein expression analyzed by Western blot.

Variables	C (*n* = 9)	PO (*n* = 9)	MO (*n* = 9)	*p* Value
Type I collagen	1.022 ± 0.199	1.026 ± 0.167	1.026 ± 0.229	0.999
TIMP-2	1.079 ± 0.372	1.223 ± 0.548	0.902 ± 0.325	0.297
TNF-α	0.682 ± 0.321	0.767 ± 0.389	0.654 ± 0.280	0.759
MMP-9	1.004 (0.883–1.246)	1.098 (0.821–1.450)	1.119 (0.859–1.472)	0.647
TIMP-4	1.000 (0.839–1.115)	1.081 (1.007–1.179)	1.016 (0.823–1.229)	0.588

C: control; PO: Pera orange juice; MO: Moro orange juice; TIMP-2: tissue inhibitor of metalloproteinase-2; TNF-α: tumor necrosis factor alpha; MMP-9: matrix metalloproteinase-9; TIMP: tissue inhibitor of metalloproteinase. Data are expressed as mean ± SD or median and 25th and 75th percentiles, in arbitrary units; ANOVA and Tukey.

## Data Availability

The datasets used and/or analyzed during the current study are available from the corresponding author on reasonable request. The data are not publicly available due to privacy.

## Supplementary Materials

The following supporting information can be downloaded at: https://www.mdpi.com/article/10.3390/metabo13080902/s1, Figure S1: Analysis of Variable Importance in Projection (VIP) Score.

## References

[1] Tsao C.W., Aday A.W., Almarzooq Z.I., Anderson C.A.M., Arora P., Avery C.L., Baker-Smith C.M., Beaton A.Z., Boehme A.K., Buxton A.E. (2023). Heart disease and stroke statistics—2023 update: A report from the American Heart Association. Circulation.

[2] Winnige P., Vysoky R., Dosbaba F., Batalik L. (2021). Cardiac rehabilitation and its essential role in the secondary prevention of cardiovascular diseases. World J. Clin. Cases.

[3] Savarese G., Becher P.M., Lund L.H., Seferovic P., Rosano G.M.C., Coats A.J.S. (2022). Global burden of heart failure: A comprehensive and updated review of epidemiology. Cardiovasc. Res..

[4] Martins D., Garcia L.R., Queiroz D.A.R., Lazzarin T., Tonon C.R., Balin P.d.S., Polegato B.F., de Paiva S.A.R., Azevedo P.S., Minicucci M.F. (2022). Oxidative Stress as a Therapeutic Target of Cardiac Remodeling. Antioxidants.

[5] Azevedo P.S., Polegato B.F., Minicucci M.F., Paiva S.A.R., Zornoff L.A.M. (2016). Cardiac remodeling: Concepts, clinical impact, pathophysiological mechanisms and pharmacologic treatment. Arq. Bras. Cardiol..

[6] Stewart J., Manmathan G., Wilkinson P. (2017). Primary prevention of cardiovascular disease: A review of contemporary guidance and literature. JRSM Cardiovasc. Dis..

[7] Cassidy A., Minihane A.-M. (2017). The role of metabolism (and the microbiome) in defining the clinical efficacy of dietary flavonoids. Am. J. Clin. Nutr..

[8] Liu R.H. (2013). Dietary bioactive compounds and their health implications. J. Food Sci..

[9] Mesquita E., Monteiro M. (2018). Simultaneous HPLC determination of flavonoids and phenolic acids profile in Pêra-Rio orange juice. Food Res. Int. Ott. Ont.

[10] Medina-Remón A., Estruch R., Tresserra-Rimbau A., Vallverdú-Queralt A., Lamuela-Raventos R.M. (2013). The effect of polyphenol consumption on blood pressure. Mini Rev. Med. Chem..

[11] Tripoli E., Guardia M.L., Giammanco S., Majo D.D., Giammanco M. (2007). Citrus flavonoids: Molecular structure, biological activity and nutritional properties: A review. Food Chem..

[12] Lazzarin T., Garcia L.R., Martins D., Queiroz D.A.R., Tonon C.R., Balin P.d.S., Polegato B.F., Paiva S.A.R.d., Azevedo P.S., Minicucci M. (2022). Role of nutrients and foods in attenuation of cardiac remodeling through oxidative stress pathways. Antioxidants.

[13] Ribeiro A.P.D., Pereira A.G., Todo M.C., Fujimori A.S.S., dos Santos P.P., Dantas D., Fernandes A.A., Zanati S.G., Hassimotto N.M.A., Zornoff L.A.M. (2021). Pera orange (*Citrus sinensis*) and Moro orange (*Citrus sinensis* (L.) Osbeck) juices attenuate left ventricular dysfunction and oxidative stress and improve myocardial energy metabolism in acute doxorubicin-induced cardiotoxicity in rats. Nutrition.

[14] Oliveira B.C., Santos P.P., Figueiredo A.M., Rafacho B.P.M., Ishikawa L., Zanati S.G., Fernandes A.A.H., Azevedo P.S., Polegato B.F., Zornoff L.A.M. (2021). Influência do Consumo de Suco de Laranja (*Citrus Sinensis*) na Remodelação Cardíaca de Ratos Submetidos a Infarto do Miocárdio. Arq. Bras. Cardiol..

[15] Daher C.F., Abou-Khalil J., Baroody G.M. (2005). Effect of acute and chronic grapefruit, orange, and pineapple juice intake on blood lipid profile in normolipidemic rat. Med. Sci. Monit. Int. Med. J. Exp. Clin. Res..

[16] Alhabeeb H., Sohouli M.H., Lari A., Fatahi S., Shidfar F., Alomar O., Salem H., Al-Badawi I.A., Abu-Zaid A. (2022). Impact of orange juice consumption on cardiovascular disease risk factors: A systematic review and meta-analysis of randomized-controlled trials. Crit. Rev. Food Sci. Nutr..

[17] Pla-Pagà L., Valls R.M., Pedret A., Calderón-Pérez L., Llauradó E., Companys J., Domenech-Coca C., Canela N., del Bas J.M., Caimari A. (2021). Effect of the consumption of hesperidin in orange juice on the transcriptomic profile of subjects with elevated blood pressure and stage 1 hypertension: A randomized controlled trial (CITRUS study). Clin. Nutr..

[18] Salamone F., Li Volti G., Titta L., Puzzo L., Barbagallo I., La Delia F., Zelber-Sagi S., Malaguarnera M., Pelicci P.G., Giorgio M. (2012). Moro orange juice prevents fatty liver in mice. World J. Gastroenterol..

[19] Cardile V., Graziano A.C.E., Venditti A. (2015). Clinical evaluation of Moro (*Citrus sinensis* (L.) Osbeck) orange juice supplementation for the weight management. Nat. Prod. Res..

[20] Barreca D., Bellocco E., Leuzzi U., Gattuso G. (2014). First evidence of C- and O-glycosyl flavone in blood orange (*Citrus sinensis* (L.) Osbeck) juice and their influence on antioxidant properties. Food Chem..

[21] Koulman A., Volmer D.A. (2008). Perspectives for metabolomics in human nutrition: An overview. Nutr. Bull..

[22] Aderemi A.V., Ayeleso A.O., Oyedapo O.O., Mukwevho E. (2021). Metabolomics: A scoping review of its role as a tool for disease biomarker discovery in selected non-communicable diseases. Metabolites.

[23] Tebani A., Bekri S. (2019). Paving the way to precision nutrition through metabolomics. Front. Nutr..

[24] Moreira V., Brasili E., Fiamoncini J., Marini F., Miccheli A., Daniel H., Lee J.J.H., Hassimotto N.M.A., Lajolo F.M. (2018). Orange juice affects acylcarnitine metabolism in healthy volunteers as revealed by a mass-spectrometry based metabolomics approach. Food Res. Int..

[25] Rangel-Huerta O.D., Aguilera C.M., Perez-de-la-Cruz A., Vallejo F., Tomas-Barberan F., Gil A., Mesa M.D. (2017). A serum metabolomics-driven approach predicts orange juice consumption and its impact on oxidative stress and inflammation in subjects from the BIONAOS study. Mol. Nutr. Food Res..

[26] Melo C.F.O.R., Delafiori J., de Oliveira D.N., Guerreiro T.M., Esteves C.Z., Lima E.d.O., Pando-Robles V., Catharino R.R. (2017). Serum metabolic alterations upon Zika infection. Front. Microbiol..

[27] Reyes D.R.A., Gomes M.J., Rosa C.M., Pagan L.U., Damatto F.C., Damatto R.L., Depra I., Campos D.H.S., Fernandez A.A.H., Martinez P.F. (2017). N-acetylcysteine influence on oxidative stress and cardiac remodeling in rats during transition from compensated left ventricular hypertrophy to heart failure. Cell. Physiol. Biochem..

[28] Bradford M.M. (1976). A rapid and sensitive method for the quantitation of microgram quantities of protein utilizing the principle of protein-dye binding. Anal. Biochem..

[29] Kologrivova I., Shtatolkina M., Suslova T., Ryabov V. (2021). Cells of the immune system in cardiac remodeling: Main players in resolution of inflammation and repair after myocardial infarction. Front. Immunol..

[30] Burke R.M., Burgos Villar K.N., Small E.M. (2021). Fibroblast contributions to ischemic cardiac remodeling. Cell. Signal..

[31] Spinale F.G. (2007). Myocardial matrix remodeling and the matrix metalloproteinases: Influence on cardiac form and function. Physiol. Rev..

[32] Vanhoutte D., Heymans S. (2010). TIMPs and cardiac remodeling: ‘Embracing the MMP-independent-side of the family’. J. Mol. Cell. Cardiol..

[33] Li L., Zhao Q., Kong W. (2018). Extracellular matrix remodeling and cardiac fibrosis. Matrix Biol..

[34] Anwar S., Khan S., Shamsi A., Anjum F., Shafie A., Islam A., Ahmad F., Hassan M.I. (2021). Structure-based investigation of MARK4 inhibitory potential of Naringenin for therapeutic management of cancer and neurodegenerative diseases. J. Cell. Biochem..

[35] Li S.-H., Wang M.-S., Ke W.-L., Wang M.-R. (2021). Naringenin alleviates myocardial ischemia reperfusion injury by enhancing the myocardial miR-126-PI3K/AKT axis in streptozotocin-induced diabetic rats. Exp. Ther. Med..

[36] Mandour D.A., Bendary M.A., Alsemeh A.E. (2021). Histological and imunohistochemical alterations of hippocampus and prefrontal cortex in a rat model of Alzheimer like-disease with a preferential role of the flavonoid “hesperidin”. J. Mol. Histol..

[37] Li J., Wang T., Liu P., Yang F., Wang X., Zheng W., Sun W. (2021). Hesperetin ameliorates hepatic oxidative stress and inflammation via the PI3K/AKT-Nrf2-ARE pathway in oleic acid-induced HepG2 cells and a rat model of high-fat diet-induced NAFLD. Food Funct..

[38] Huang S.M., Bisogno T., Petros T.J., Chang S.Y., Zavitsanos P.A., Zipkin R.E., Sivakumar R., Coop A., Maeda D.Y., De Petrocellis L. (2001). Identification of a new class of molecules, the arachidonyl amino acids, and characterization of one member that inhibits pain. J. Biol. Chem..

[39] Burstein S.H., Adams J.K., Bradshaw H.B., Fraioli C., Rossetti R.G., Salmonsen R.A., Shaw J.W., Walker J.M., Zipkin R.E., Zurier R.B. (2007). Potential anti-inflammatory actions of the elmiric (lipoamino) acids. Bioorg. Med. Chem..

[40] Azevedo P.S., Minicucci M.F., Santos P.P., Paiva S.A.R., Zornoff L.A.M. (2013). Energy metabolism in cardiac remodeling and heart failure. Cardiol. Rev..

[41] Gómez-Fernández J.C., Corbalán-García S. (2007). Diacylglycerols, multivalent membrane modulators. Chem. Phys. Lipids.

[42] Alwarawrah M., Hussain F., Huang J. (2016). Alteration of lipid membrane structure and dynamics by diacylglycerols with unsaturated chains. Biochim. Biophys. Acta BBA-Biomembr..

[43] Samouillan V., Martinez de Lejarza Samper I.M., Benitez Amaro A., Vilades D., Dandurand J., Casas J., Jorge E., de Gonzalo Calvo D., Gallardo A., Lerma E. (2020). Biophysical and lipidomic biomarkers of cardiac remodeling post-myocardial infarction in humans. Biomolecules.

[44] Kanehisa M., Goto S. (2000). KEGG: Kyoto encyclopedia of genes and genomes. Nucleic Acids Res..

[45] Kanehisa M., Sato Y., Furumichi M., Morishima K., Tanabe M. (2019). New approach for understanding genome variations in KEGG. Nucleic Acids Res..

[46] Sharma A., Madan N. (2019). Role of niacin in current clinical practice. Minerva Med..

[47] Böger R.H. (2008). L-Arginine therapy in cardiovascular pathologies: Beneficial or dangerous?. Curr. Opin. Clin. Nutr. Metab. Care.

[48] O’Sullivan S., Medina C., Ledwidge M., Radomski M.W., Gilmer J.F. (2014). Nitric oxide-matrix metaloproteinase-9 interactions: Biological and pharmacological significance—NO and MMP-9 interactions. Biochim. Biophys. Acta.

[49] Chaya N., Terauchi K., Yamagata Y., Kinjo J., Okabe H. (2004). Antiproliferative Constituents in Plants 14.1 Coumarins and Acridone Alkaloids from *Boenninghausenia japonica* NAKAI. Biol. Pharm. Bull..

[50] Detsi A., Kontogiorgis C., Hadjipavlou-Litina D. (2017). Coumarin derivatives: An updated patent review (2015–2016). Expert Opin. Ther. Pat..

[51] Wishart D.S., Feunang Y.D., Marcu A., Guo A.C., Liang K., Vázquez-Fresno R., Sajed T., Johnson D., Li C., Karu N. (2018). HMDB 4.0: The human metabolome database for 2018. Nucleic Acids Res..

[52] Sahu D., Raghav S.K., Gautam H., Das H.R. (2015). A novel coumarin derivative, 8-methoxy chromen-2-one alleviates collagen induced arthritis by down regulating nitric oxide, NFκB and proinflammatory cytokines. Int. Immunopharmacol..

[53] Hauser F., Li Z., Waadt R., Schroeder J.I. (2017). SnapShot: Abscisic Acid Signaling. Cell.

[54] Koyama R., Roberto S.R., de Souza R.T., Borges W.F.S., Anderson M., Waterhouse A.L., Cantu D., Fidelibus M.W., Blanco-Ulate B. (2018). Exogenous abscisic acid promotes anthocyanin biosynthesis and increased expression of flavonoid synthesis genes in *Vitis vinifera* × *Vitis labrusca* table grapes in a subtropical region. Front. Plant Sci..

[55] Ferrero M., Pagliarani C., Novák O., Ferrandino A., Cardinale F., Visentin I., Schubert A. (2018). Exogenous strigolactone interacts with abscisic acid-mediated accumulation of anthocyanins in grapevine berries. J. Exp. Bot..

[56] Fujiwara Y. (2008). Cyclic phosphatidic acid-a unique bioactive phospholipid. Biochim. Biophys. Acta.

[57] Yamamoto S., Yamashina K., Ishikawa M., Gotoh M., Yagishita S., Iwasa K., Maruyama K., Murakami-Murofushi K., Yoshikawa K. (2017). Protective and therapeutic role of 2-carba-cyclic phosphatidic acid in demyelinating disease. J. Neuroinflammation.

[58] Murakami-Murofushi K., Uchiyama A., Fujiwara Y., Kobayashi T., Kobayashi S., Mukai M., Murofushi H., Tigyi G. (2002). Biological functions of a novel lipid mediator, cyclic phosphatidic acid. Biochim. Biophys. Acta.

[59] Maeda-Sano K., Gotoh M., Morohoshi T., Someya T., Murofushi H., Murakami-Murofushi K. (2014). Cyclic phosphatidic acid and lysophosphatidic acid induce hyaluronic acid synthesis via CREB transcription factor regulation in human skin fibroblasts. Biochim. Biophys. Acta.

[60] Gotoh M., Nagano A., Tsukahara R., Murofushi H., Morohoshi T., Otsuka K., Murakami-Murofushi K. (2014). Cyclic phosphatidic acid relieves osteoarthritis symptoms. Mol. Pain.

[61] Litwiniuk M., Krejner A., Speyrer M.S., Gauto A.R., Grzela T. (2016). Hyaluronic Acid in Inflammation and Tissue Regeneration. Wounds Compend. Clin. Res. Pract..

[62] Liu Y.-W., Hao Y.-C., Chen Y.-J., Yin S.-Y., Zhang M.-Y., Kong L., Wang T.-Y. (2018). Protective effects of sarsasapogenin against early stage of diabetic nephropathy in rats. Phytother. Res. PTR.

[63] National Center for Biotechnology Information PubChem Compound Summary for CID 12313943, Torvoside C. https://pubchem.ncbi.nlm.nih.gov/compound/Torvoside-C.

[64] Chen P.-S., Shih Y.-W., Huang H.-C., Cheng H.-W. (2011). Diosgenin, a steroidal saponin, inhibits migration and invasion of human prostate cancer PC-3 cells by reducing matrix metalloproteinases expression. PLoS ONE.

[65] Wu Z., Zhao X., Miyamoto A., Zhao S., Liu C., Zheng W., Wang H. (2019). Effects of steroidal saponins extract from Ophiopogon japonicus root ameliorates doxorubicin-induced chronic heart failure by inhibiting oxidative stress and inflammatory response. Pharm. Biol..

