# Supplementary material for: Effects of Pera Orange Juice and Moro Orange Juice in Healthy Rats: A Metabolomic Approach
Source: Metabolites. 2023 Aug 2;13(8):902. doi: 10.3390/metabo13080902 (PMC10456557; doi:10.3390/metabo13080902)
Supplement: Supplementary file 1 [file metabolites-13-00902-s001.zip › metabolites-2503731-supplementary.pdf]

Analysis of Variable Importance in Projection (VIP). Main masses of low molecular weight (100 to 700 *m/z*) responsible for discriminating between the PO group and the C group.

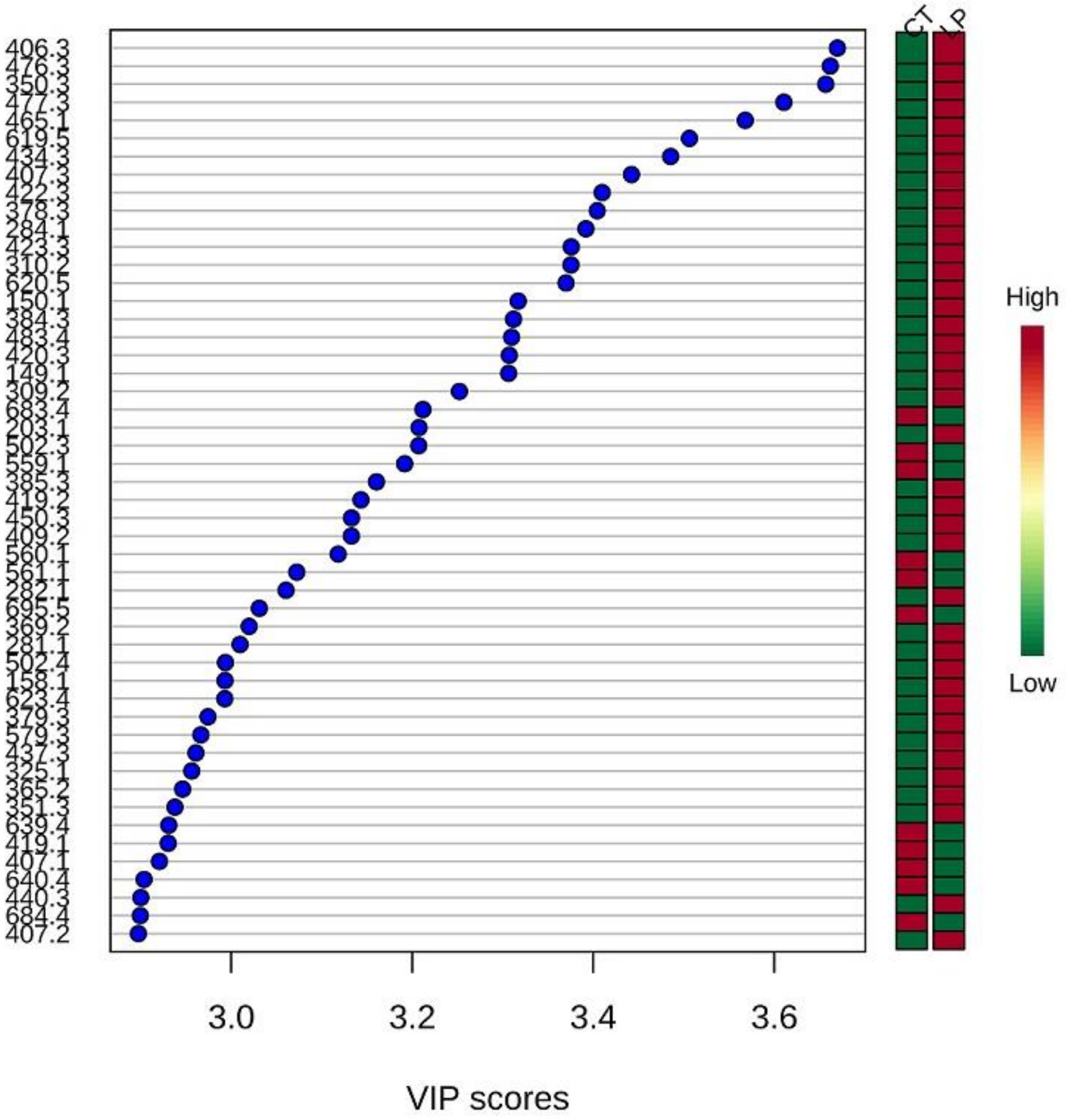

CT: Control group (C); LP: Pera Orange Group (PO)

Analysis of Variable Importance in Projection (VIP). Main masses of low molecular weight (700 a 1700 *m/z*) responsible for discriminating between the PO group and the C group.

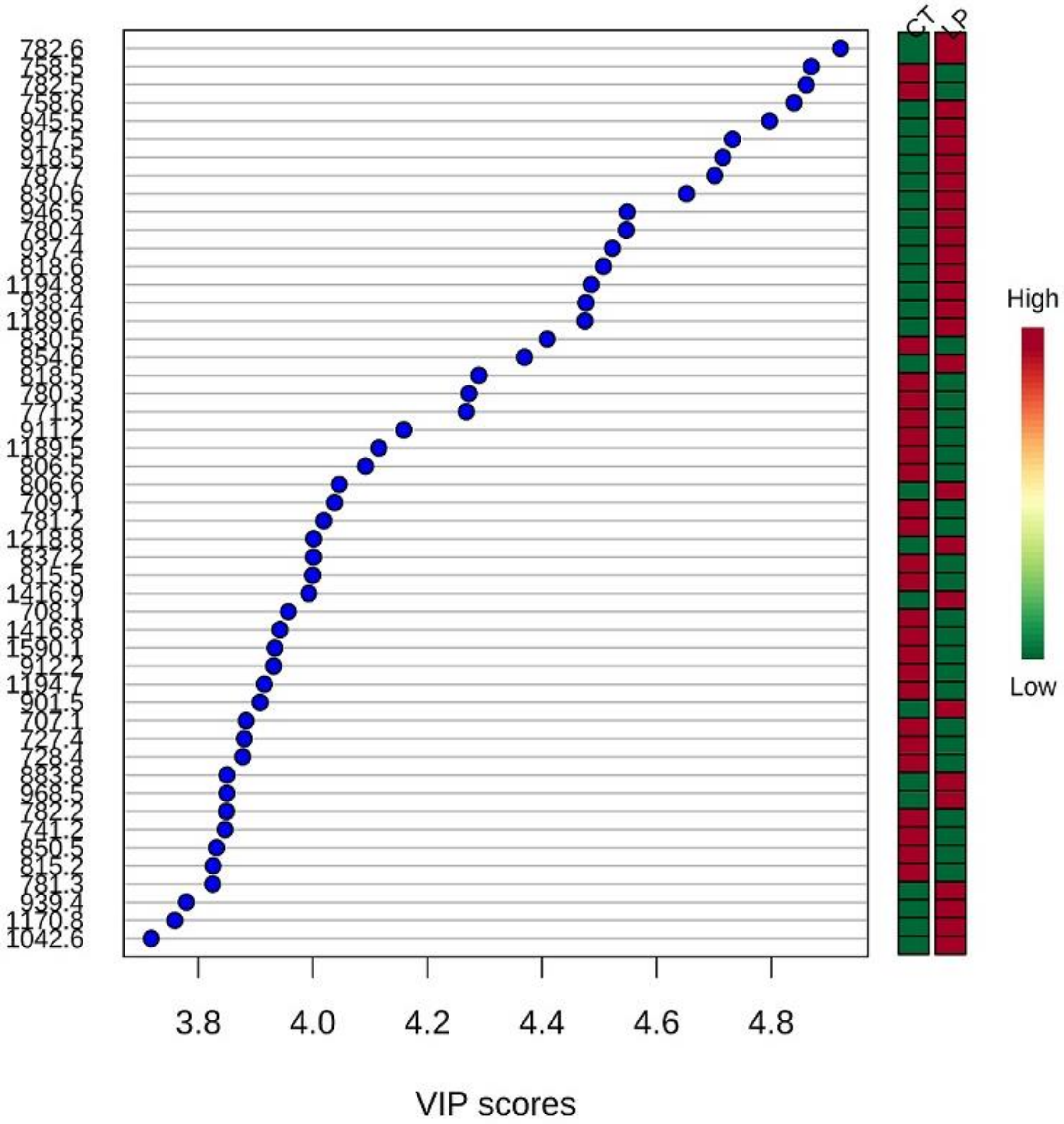

CT: Control group (C); LP: Pera Orange Group (PO)

Analysis of Variable Importance in Projection (VIP). Main masses of low molecular weight (100 to 700 *m/z*) responsible for discriminating between the MO group and the C group.

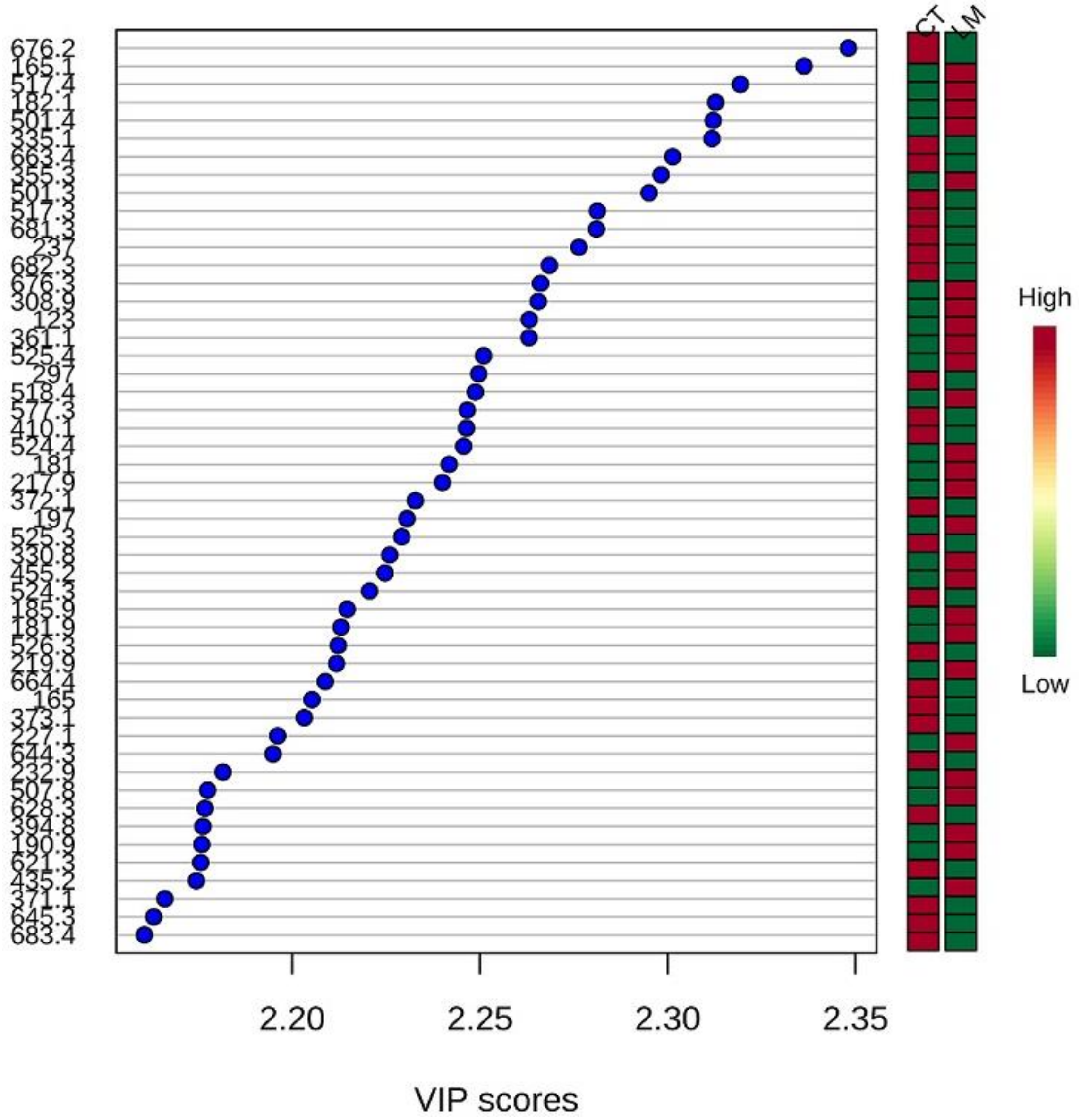

CT: Control group (C); LM: Moro Orange Group (MO)

Analysis of Variable Importance in Projection (VIP). Main masses of low molecular weight (700 a 1700 *m/z*) responsible for discriminating between the MO group and the C group.

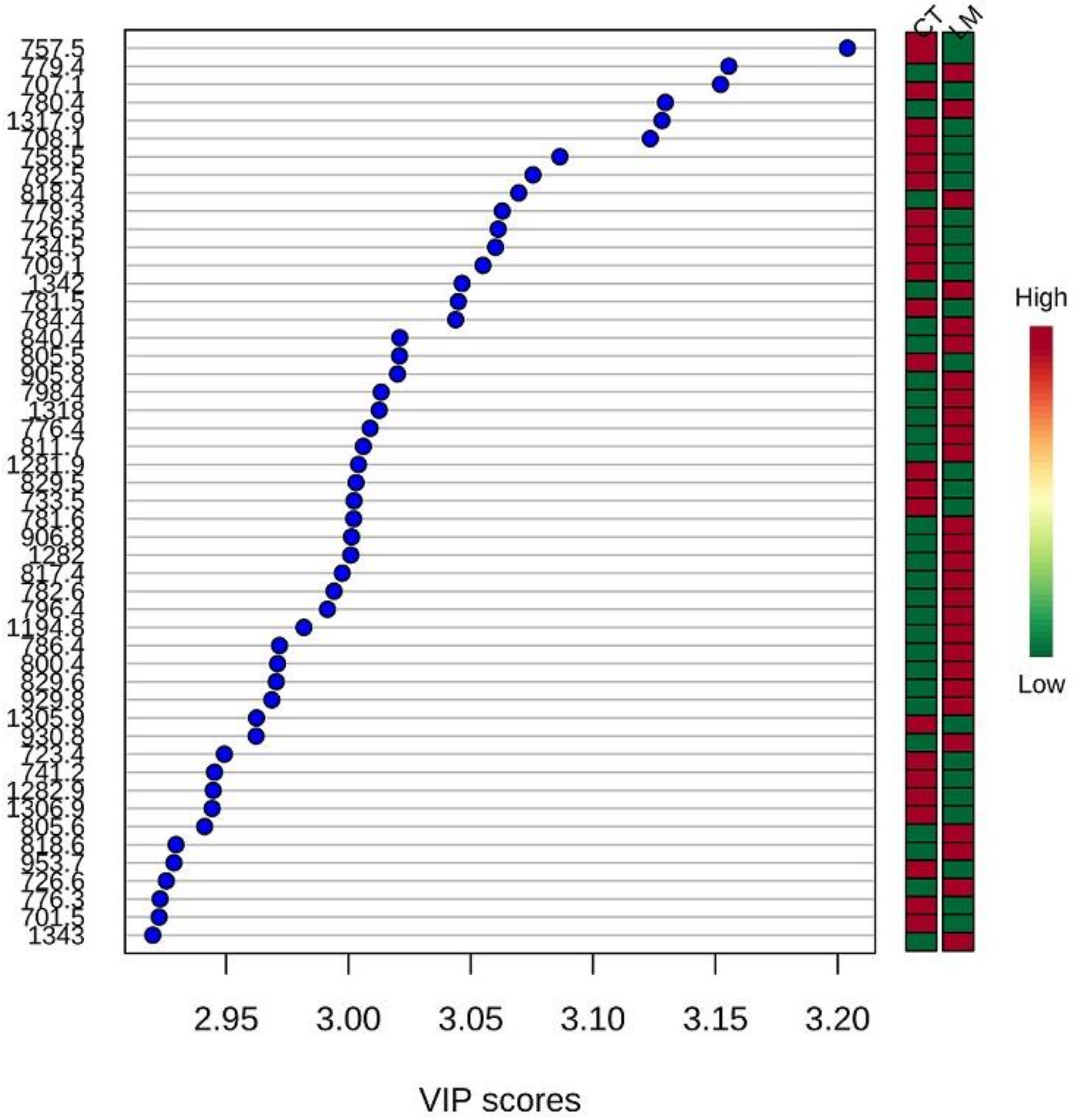

CT: Control group (C); LM: Moro Orange Group (MO)
